# Optimization of Ultrasonic-Assisted Extraction, Characterization and Antioxidant and Immunoregulatory Activities of *Arthrospira platensis* Polysaccharides

**DOI:** 10.3390/molecules29194645

**Published:** 2024-09-30

**Authors:** Na Wang, Jingyi Qin, Zishuo Chen, Jiayi Wu, Wenzhou Xiang

**Affiliations:** 1 Department of Cell Biology and Genetics, School of Basic Medical Sciences, Hengyang Medical School, University of South China, Hengyang 421001, China; 2 Institute of Cytology and Genetics, School of Basic Medical Sciences, Hengyang Medical School, University of South China, Hengyang 421001, China; 3 CAS Key Laboratory of Tropical Marine Bio-Resources and Ecology, Guangdong Key Laboratory of Marine Materia Medica, Institution of South China Sea Ecology and Environmental Engineering, RNAM Center for Marine Microbiology, South China Sea Institute of Oceanology, Chinese Academy of Sciences, Guangzhou 510301, China; 4 University of Chinese Academy of Sciences, Beijing 100049, China; 5 Greater Bay Area Institute of Precision Medicine (Guangzhou), Guangzhou 511466, China

**Keywords:** *Arthrospira platensis*, polysaccharides, ultrasonic-assisted extraction, characterization, antioxidant, immunoregulatory

## Abstract

This study aimed to enhance the ultrasonic-assisted extraction (UAE) yield of seawater *Arthrospira platensis* polysaccharides (APPs) and investigate its structural characteristics and bioactivities. The optimization of UAE achieved a maximum crude polysaccharides yield of 14.78%. The optimal extraction conditions were a liquid–solid ratio of 30.00 mL/g, extraction temperature of 81 °C, ultrasonic power at 92 W and extraction time at 30 min. After purification through cellulose DEAE-52 and Sephadex G-100 columns, two polysaccharide elutions (APP-1 and APP-2) were obtained. APP-2 had stronger antioxidant and immunoregulatory activities than APP-1, thus the characterization of APP-2 was conducted. APP-2 was an acidic polysaccharide consisting of rhamnose, glucose, mannose and glucuronic acid at a ratio of 1.00:24.21:7.63:1.53. It possessed a molecular weight of 72.48 kDa. Additionally, APP-2 had linear and irregular spherical particles and amorphous structures, which contained pyranoid polysaccharides with alpha/beta glycosidic bonds. These findings offered the foundation for APP-2 as an antioxidant and immunomodulator applied in the food, pharmaceutical and cosmetic industries.

## 1. Introduction

Polysaccharides, a common variety of macromolecular compounds, consist of more than 10 monosaccharides, as well as a backbone and a few side chains linked by glycosidic bonds [1]. Numerous studies have confirmed that polysaccharides are ubiquitously found in plants, animals, microorganisms and microalgae with a variety of biological activities [2]. Particularly, microalgae polysaccharides have raised wide attention attributed to their novelty, easy modification, biodegradability, safety and high activity, so they remain as hot spots in the functional food, pharmaceutical and cosmetics industries [3]. At present, it is worth pointing out that polysaccharides extracted from microalgae have been suggested to possess potential antioxidant and immunomodulatory activities, including scavenging free radicals, restraining lipid oxidation, activating macrophages and releasing various cytokines [4]. The relationship between the antioxidant and immunoregulatory activities of polysaccharides is generally closely intertwined. Polysaccharide antioxidants eliminate excess reactive oxygen species (ROS) and mitigate oxidative damage, thereby maintaining the balance of the immune system [5]. Conversely, immunomodulation bolsters the body’s defense mechanisms, aiding in resisting oxidative stress. Therefore, it is urgent to explore novel bioactive polysaccharides from microalgae with antioxidant and immune-enhancing properties.

*Arthrospira platensis*, commercialized as *Spirulina platensis*, is a blue-green microalga that is characterized by its wide geographic distribution, strong adaptability and fast growth [6]. More importantly, *Arthrospira platensis* was not only regarded as a food source for the future by the International Association of Applied Microbiology, but also certified as safe for human consumption by the United States Food and Drug Administration [7]. In addition to being an ideal superfood launched into the market, *Arthrospira platensis* is also considered to be extensively applied in pharmaceutical, animal feed additives, cosmetics, biofuel and agricultural industries; this is because *Arthrospira platensis* is rich in various high-value nutrients, such as polysaccharides [8]. *Arthrospira platensis* polysaccharides (APPs) were reported to exhibit various biological activities, including antioxidant, anti-tumor, antiviral, anticoagulant, antidiabetic and immunoregulatory activities and so on [9,10]. The sulfated polysaccharides derived from *Arthrospira* platensis using alkali extraction were found to exhibit potent antioxidant activity in 2,2-diphenyl-1-picrylhydrazyl (DPPH), hydrogen peroxide, hydroxyl free radical scavenging and nitric oxide (NO) scavenging [11]. In addition to exhibiting in vitro antioxidant effects, hot water extracted APPs were found to have beneficial effects on oxidative stress in vivo by modulating microRNAs and gut microbiota in *Caenorhabditis elegans* [12]. A hot water extraction heteropolysaccharide from *Arthrospira platensis* showed good immune-enhancing activity on RAW264.7 macrophages and significantly inhibited the growth of A549 lung cancer cells [13]. *Spirulina platensis* polysaccharides (PSP) and its fractions (PSP-1, PSP-2, PSP-3, PSP-L and PSP-M) showed varying degrees of immunomodulatory activities in RAW 264.7 cells via activating the MAPK and nuclear factor-κB (NF-κB) pathways by the TLR4 receptor [14]. As mentioned above, different *Arthrospira platensis* sources or extraction methods may cause different bioactivities of APP.

*Arthrospira platensis* can be cultured in both freshwater and seawater, among which the strain cultured in seawater gained a great deal of attention, resulting from natural seawater improving the quality of the active products, increasing the resistance of the cell and effectively avoiding the enrichment of heavy metal ions and overweight. Recent literature confirmed that seawater cultivation not only increased the carbohydrate production of *Arthrospira platensis* but also was beneficial for improving its biological activity, with a similar phenomenon observed in a previous study [15,16]. Hence, taking seawater *Arthrospira platensis* as the target material for polysaccharide extraction was of great significance.

Currently, the conventional techniques for polysaccharide extraction encompass hot water, acid, alkali, and enzymatic extraction. However, these approaches suffer from drawbacks including low yield and efficiency, lengthy extraction time and high cost, as well as the degradation and denaturation of the polysaccharide structure, resulting in the limitation of industrial utilization in polysaccharides [17]. Therefore, modern auxiliary methods, such as ultrasonic-assisted extraction (UAE), microwave-assisted extraction and supercritical fluid extraction have appeared. Among them, the UAE has gained obvious attention due to its simplicity, swiftness, higher yield and lower energy consumption attributes, suitable for large-scale industry applications [18]. Ultrasonic waves can produce a cavitation effect that disrupts cell walls via the increase in temperature and pressure, accelerating the dissolution and release of the target intracellular polysaccharides from materials thus enhancing the yield [19]. In addition, proper ultrasound treatment can alter the physiochemical properties of polysaccharides, including the molecular weight (Mw) and viscosity, contributing to remarkable bioactivities [20]. As reported, the antioxidant activity of *Panax notoginseng* flower polysaccharides extracted by UAE was enhanced compared with heat reflux extraction, as well as the yields, the contents of uronic acid and total phenol, etc. [21]. Furthermore, UAE has been demonstrated to greatly increase the scavenging activities against DPPH and hydroxyl radicals, and the immunomodulatory activity of *Nostoc commune* Vaucher polysaccharides [22]. Response surface methodology (RSM) is a tool utilized for optimizing the extraction process, evaluating the impacts of process parameters and their interactions on the extraction yield, and predicting the optimal extraction conditions to enhance the overall extraction efficiency [23]. Additionally, RSM offers the advantages of high efficiency, accuracy and resource conservation. Therefore, optimizing the extraction conditions of polysaccharides holds immense importance for large-scale implementation in industries.

As far as we know, there was no systematic reference reported on the optimization of the UAE of seawater *Arthrospira platensis* polysaccharides and the evaluation of its structure and antioxidant and immunoregulatory activity, hindering the exploitation and utilization of valuable resources. Therefore, this research adopted RSM and Box–Behnken design (BBD) to optimize the conditions for maximizing the UAE yield of APP. After purification by Cellulose DEAE-52 and a Sephadex G-100 gel-filtration column, the antioxidant and immunoregulatory potentials of the two elutions were, respectively, investigated using DPPH, 2,2-azino-bis 3-ethylbenzthiazoline-6-sulphonic acid (ABTS), superoxide free radical scavenging and ferrous-chelating activity, as well as a RAW 264.7 cell. Furthermore, the polysaccharide fraction exhibiting significant activity was characterized using high-performance size-exclusion chromatography (HPSEC), high-performance anion-exchange chromatography (HPAEC), ultraviolet–visible (UV–Vis) spectroscopy, Fourier transform infrared spectroscopy (FT-IR), X-ray diffractometry (XRD), scanning electron microscopy (SEM) and atomic force microscopy (AFM). This work supplied scientific evidence for the further research and development of APP in medicine and functional food applications.

## 2. Results and Discussion

### 2.1. Effects of UAE Parameters on APP Yield

**Figure 1:**
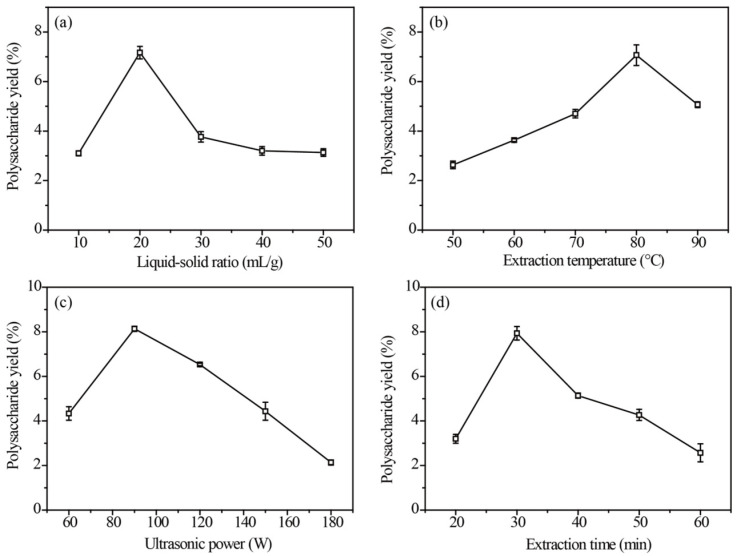
Effects of liquid–solid ratio (**a**), extraction temperature (**b**), ultrasonic power (**c**) and extraction time (**d**) on the extraction yield of *Arthrospira platensis* polysaccharides (n = 3).

The extraction yield reached its highest point (7.21%) at the liquid–solid ratio of 20 mL/g (Figure 1a). Subsequently, as the ratio increased, the yield gradually decreased due to excess water causing raw material overswelling. This phenomenon could be attributed to the polysaccharide reaching its maximum mass transfer driving force [24]. Accordingly, liquid–solid ratios of 10, 20 and 30 mL/g were selected for the BBD experiment.

The effect of varying extraction temperatures on APP yield is illustrated in Figure 1b. The extraction temperature ranging from 50 to 80 °C was favorable for polysaccharide extraction, with a gradual decrease observed as the temperature increased. The solubility of polysaccharides in the extract was enhanced by high temperatures, facilitating their diffusion from cells into the solution. However, elevated temperatures also result in a reduction in the viscosity of the extract, thereby increasing its vapor pressure [20]. Therefore, extraction temperatures at 70, 80 and 90 °C were selected for the BBD design.

Figure 1c depicts the effects of the ultrasonic power on APP. The results demonstrated that an ultrasonic power range of 60–90 W was optimal for polysaccharide extraction. However, as the ultrasonic power increased, there was a decrease in the yield of extracted polysaccharides. It was observed that an appropriate mechanical force generated by ultrasound facilitated the dissolution and release of APP, while excessive force led to degradation and consequently reduced the extraction yield [18]. Based on these results, the ultrasonic power at 60, 90 and 120 W was selected for the BBD design.

As shown in Figure 1d, the largest APP yield of 7.86% was obtained at 30 min. However, as the extraction time further increased to 40, 50 or 60 min, the APP yield decreased. A shorter extraction time was unfavorable for adequate polysaccharide solubilization, while a prolonged extraction time led to the degradation of polysaccharide molecules [25]. Thus, the extraction time of 20, 30 and 40 min was selected for the BBD experiment.

### 2.2. Response Surface Optimization on UAE Conditions of APP

RSM is an important statistical and computational technique that can be adopted to determine the influence of each factor and their interaction on the extraction rate to identify optimal conditions. It has the advantages of fewer tests and low cost and time [23]. A 27-run BBD was designed and fitted via polynomial regression (Table 1). The relationship between APP yield (Y) and the four extraction parameters (A–D) was demonstrated in the coded level by the following equation:

Polysaccharide yield (%) = 14.10 + 0.16A + 0.69B + 0.57C − 0.24D − 0.15AB − 0.33AC + 0.10AD + 0.25BC + 1.07BD + 1.35CD + 0.52A^2^ − 3.18B^2^ − 2.63C^2^ − 1.53D^2^ (1)

The analysis of variance for the regression model on the polysaccharides extraction rate from *Arthrospira platensis* is presented in Table 2. The model demonstrated high statistical significance with low *p*-values (<0.0001) and accurately predicted the APP yield by an insignificant difference in the Lack of Fit (*p* > 0.05), thereby establishing its reliability. The values of the coefficient of variance (C.V.), the regression coefficient (R^2^) and the adjusted coefficient of determination (Adj-R^2^) were 6.33%, 0.9526 and 0.9002, respectively, further confirming the credibility of experimental data [18]. Moreover, the “Adeq Precision” was 13.066, which was greater than four, indicating that the signal was sufficient and the model could be used in the navigation of design space.

**Figure 2:**
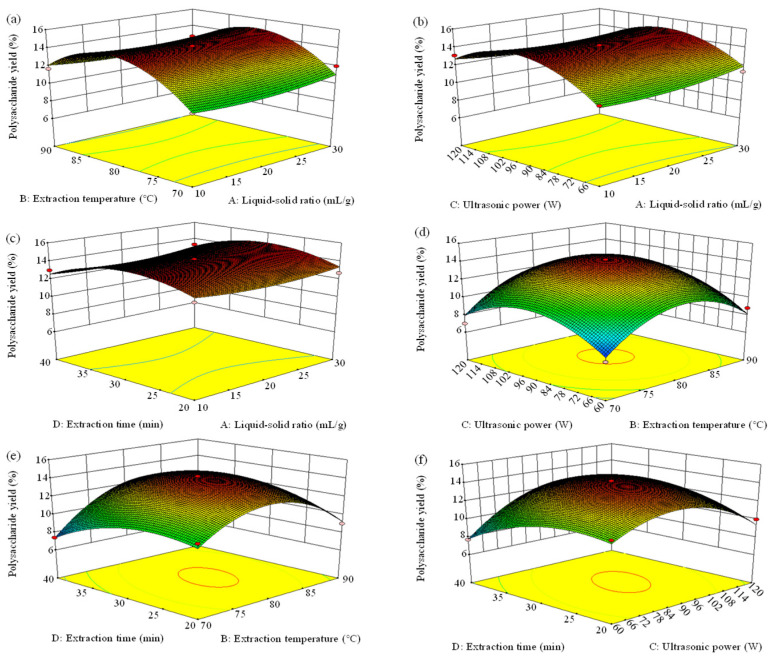
3D response surface plots (**a**–**f**) of the interaction effects of variables (A: liquid–solid ratio, mL/g; B: extraction temperature, °C; C: ultrasonic power, W; and D: extraction time, min) on *Arthrospira platensis* polysaccharide yield.

**Figure 3:**
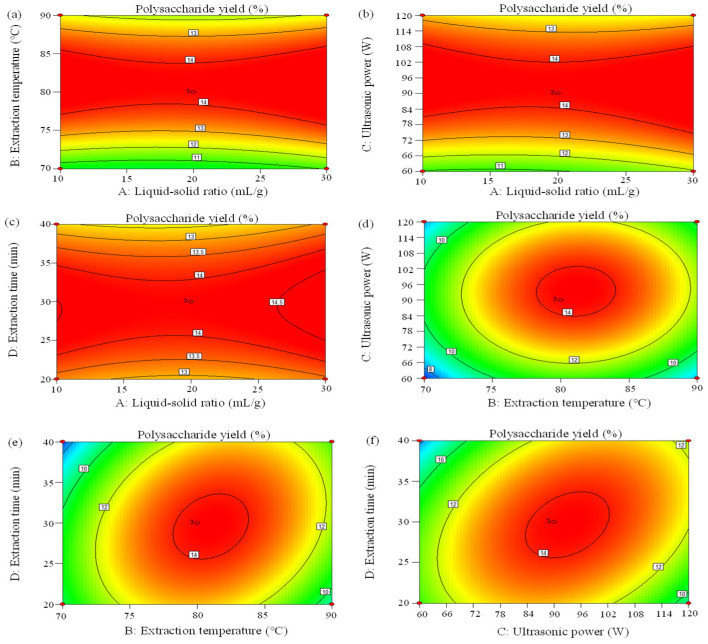
Contour plots (**a**–**f**) showing the effect of variables (A: liquid–solid ratio, mL/g; B: extraction temperature, °C; C: ultrasonic power, W; and D: extraction time, min) on the extraction yield of *Arthrospira platensis* polysaccharides. The number of contour lines indicates the elevation of the location. A denser arrangement of contour lines signifies significant changes in the terrain, whereas a sparser arrangement suggests relatively flat topography.

The significance of each coefficient was tested using F-value and *p*-value. The analysis of variance indicated that B, C, BD, CD, B^2^, C^2^, and D^2^ were significant model terms. The order of the influence of four independent variables on APP extraction yield ranked as B > C > D > A based on F-value, manifesting that extraction temperature (B) had the largest impact (*p* < 0.01) on yield, whereas the liquid–solid ratio (A) had an insignificant impact (*p* > 0.05). The three-dimensional response surface plots (Figure 2) and two-dimensional response surface plots (Figure 3) performed the effects of interacting variables on APP yield. In general, the steeper the surface in three-dimensional plots, the more obvious the variable impact on the APP yield was [26]. Furthermore, a faster color change indicated a greater slope, that is, a more significant effect on the results. The greater the shape deviated from a circular contour in two-dimensional plots, the more sensitive the APP yield was to the change in extraction condition. The denser the contour lines, the more obvious the interaction. In this case, it can be observed that the slopes of the curves for extraction temperature vs. extraction time (BD) and ultrasonic power vs. extraction time (CD) were larger, indicating significant impacts (*p* < 0.05), which were in line with the results of the ANOVA analysis. Instead, the remaining coefficients did not show statistical significance (*p* > 0.05).

### 2.3. Confirmation and Verification of the Predictive Model

The optimal extraction conditions of APP were established by Design-Expert Software (8.0.6) and the computed optimum parameters were as follows: liquid–solid ratio, 30.00 mL/g; extraction temperature, 80.89 °C; ultrasonic power, 91.63 W; and extraction time, 30.10 min, under which the maximum predicted extraction yield was 14.81%. To verify the model prediction, triplicate confirmatory experiments under the modified optimal condition of a liquid–solid ratio of 30.00 mL/g, extraction temperature of 81 °C, ultrasonic power of 92 W and extraction time of 30 min were carried out. The actual APP yield was 14.78%, which was closely aligned with the forecast value, confirming the validation of the response model. This model could be applied to extract polysaccharides from *Arthrospira platensis*. It is worth mentioning that the APP extraction rate of the optimum extraction method used in this work was significantly higher than that of the UAE extraction method used before with an extraction yield of 6.10%. Additionally, the optimization of hot water and alkali extraction led to the maximum crude APP yield of 4.85% [6] and 8.78% [27], respectively, which were much lower than the APP yield in our study. The combination of different extraction methods may lead to better extraction efficiency [6]. To enhance the yield, the integration of multiple techniques for APP extraction may emerge as a prospective trend in the future. Furthermore, it is imperative to devise cost-effective and environmentally sustainable methods for extracting APP.

### 2.4. Isolation and Purification of APP

**Figure 4:**
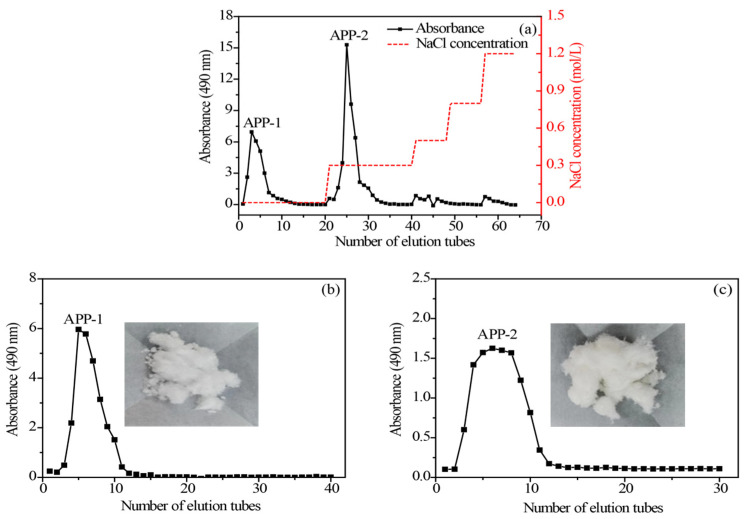
DEAE-cellulose (**a**) and Sephadex G-100 elution profiles (**b**,**c**) of polysaccharides from *Arthrospira platensis*.

The crude APP was obtained from *Arthrospira platensis* via lyophilization and UAE extraction, followed by ethanol precipitation and deproteinization. Subsequently, the APP was separated and further purified by a DEAE-52 cellulose column, obtaining two components, including the distilled water eluting fraction APP-1 (31.50% yield from APP) and the 0.3 M NaCl eluting fraction APP-2 (50.20% yield from APP), respectively (Figure 4a). To obtain high-purity polysaccharides, a Sephadex G-100 column was applied and the elution curves are illustrated in Figure 4b,c, of which each elution curve showed a single and symmetrical elution peak. Individual fractions were collected, affording APP-1 (28.94%) and APP-2 (49.72%) compared with APP, respectively.

### 2.5. Antioxidant Activity Analysis

**Figure 5:**
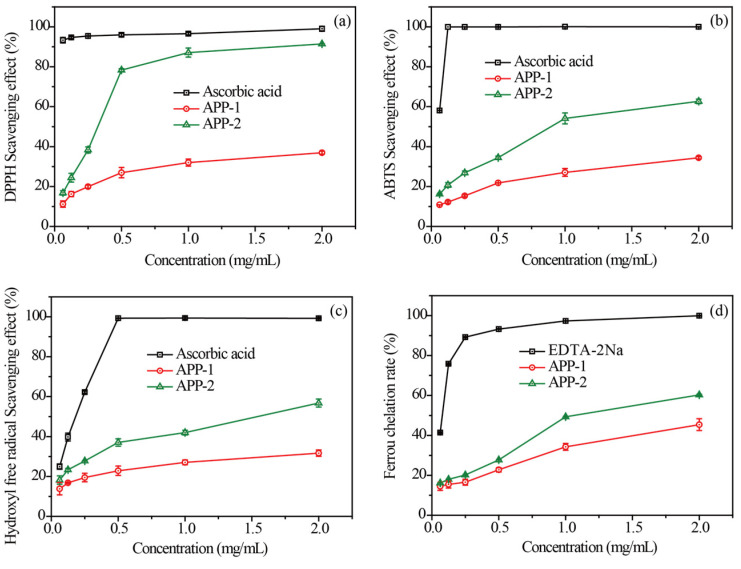
Antioxidant activities of APP-1 and APP-2. Scavenging effects against DPPH (**a**), ABTS (**b**) and Hydroxyl (**c**) free radicals, and Ferrous chelation rate (**d**).

The DPPH radical scavenging activities of APP-1, APP-2 and ascorbic acid (Vc) are illustrated in Figure 5a, in which the clearance rose as the polysaccharides concentration increased in the range of 0.0625–2.0 mg/mL. The maximum scavenging rates of APP-1 and APP-2 were 36.93% and 91.42%, respectively, at a concentration of 2 mg/mL, which were lower in comparison to 99.03% for Vc. The IC_50_ values for DPPH scavenging by APP-1 and APP-2 were 6.03 and 0.27 mg/mL, respectively (Table S1), which exhibited higher values compared to that of Vc. However, Wu et al. [28] reported that two spirulina platensis polysaccharides from different separation methods were potent in acting as antioxidants with IC_50_ values of 0.71 and 0.83 mg/mL, respectively. The IC_50_ values of Fucus vesiculosus, Gracilaria fisheri and Pinus koraiensis polysaccharides on removing DPPH were 0.97, 3.00 and 122.2 mg/mL, respectively [29,30,31]. The results showed that APP-2 showed a strong ability to scavenge DPPH radicals. It is believed that the biological activity of polysaccharides is influenced by their chemical structure, such as the molecular weight, the composition of monosaccharides, branching configuration and conformation [6]. In addition, research shows that the chemical structure of microalgae polysaccharides depends on algal species, population age, environmental conditions, geographic location, seaweed harvest season and the isolation and purification methods used [32,33].

Figure 5b demonstrates a concentration-dependent relationship between the concentration of APP and its activity in scavenging ABTS, which paralleled the correlation observed between polysaccharides’ electron transport capacity and their antioxidant activity. Herein, at 0.0625 mg/mL, the two elutions exhibited the lowest ABTS clearance rates of 10.83% and 16.23%, respectively. Conversely, at a concentration of 2 mg/mL, the ABTS clearance rates of the two elutions reach their highest values of 34.40% and 62.67%, respectively. Nevertheless, the ABTS radical scavenging rates of APP-1 and APP-2 were significantly weaker than Vc. The sulfated polysaccharides derived from Gracilaria chouae had an ABTS scavenging rate of 19.07% under a concentration of 3 mg/mL [34]. The IC_50_ values of APP-1 and APP-2 were 8.85 and 0.97 mg/mL, respectively (Table S1). Therefore, the IC_50_ of APP-2 was lower than many previously reported polysaccharides, including *Rhodosorus* sp. SCSIO-45730 (IC_50_ = 1.74 mg/mL) [33], *Botryococcus braunii* (12.83 mg/mL) [35] and *Pinus koraiensis* (IC_50_ = 23.60 mg/mL) [31].

Figure 5c showed the scavenging activities of APP-1, APP-2 and Vc on hydroxyl radicals at different concentrations, in which the scavenging effects were kept increasing with dose. The scavenging rates of the two fractions were 31.70% and 56.77%, respectively, at 2.0 mg/mL, which were lower than that of Vc (99.25%). Furthermore, APP-2 possessed a stronger scavenging effect on hydroxyl radicals as compared to APP-1 while being weaker than that of Vc based on the IC_50_ values in Table S1. Meanwhile, APP-2 showed a better scavenging activity than previously reported polysaccharides, for instance, polysaccharides fractions (HN1, HN2 and HN3) from *Nemacystus decipients* (with IC_50_ values of 4.12, 4.37 and 3.07 mg/mL) [36], *Alternaria* sp. SP-32 polysaccharides (with an IC_50_ of 4.20 mg/mL) [37] and *Holothuria fuscogliva* polysaccharides (with an IC_50_ of 3.74 mg/mL) [38].

As seen in Figure 5d, the ferrous chelation rates of the two polysaccharides and EDTA-2Na were concentration-dependent. In the concentration range of 0.0625–0.25 mg/mL, there was no significant change in the chelation rate of ferrous iron with varying concentrations. However, within the concentration range of 0.25 to 2.00 mg/mL, a substantial variation in the chelation rate of ferrous iron was observed. At the highest concentration of 2 mg/mL, APP-1 and APP-2 exhibited significantly higher activity for chelating ferrous iron (45.39% and 60.28%, respectively). Similarly, at the lowest concentration of 0.0625 mg/mL, their chelation activities towards ferrous iron were measured to be 14.48% and 16.16%, respectively. EDTA exhibited excellent chelating activity with a chelating rate close to 100% at 2.0 mg/mL. *Rhodosorus* sp. SCSIO-45730 polysaccharide had a ferrous chelating rate of approximately 43.19% at 2.0 mg/mL [33]. The IC_50_ values for APP-1, APP-2 and EDTA-2Na were determined to be 3.75, 1.23 and 0.07 mg/mL, respectively (Table S1). The results showed that APP-2 had a stronger ferrous chelating activity than APP-1.

In general, APP-2 exhibited significant antioxidant activity. Disease in almost all human systems was closely associated with oxidative stress and damage, whereas APP-2 could play an antioxidant role in reducing oxidative stress. With the continuous emergence of novel diseases, there is a growing interest in utilizing antioxidants as a means to effectively prevent and treat diseases. Under the background of the thriving development of the big health industry, the research and development of polysaccharide-based foods, pharmaceuticals and cosmetics is currently a prominent area of investigation and represents one of the market’s most crucial functional requirements [39]. Therefore, it is of great importance to develop and utilize APP-2 as a natural antioxidant.

### 2.6. Immunomodulatory Activity Analysis

#### 2.6.1. Effects of APP-1 and APP-2 on the Cell Viability of RAW 264.7 Cells

**Figure 6:**
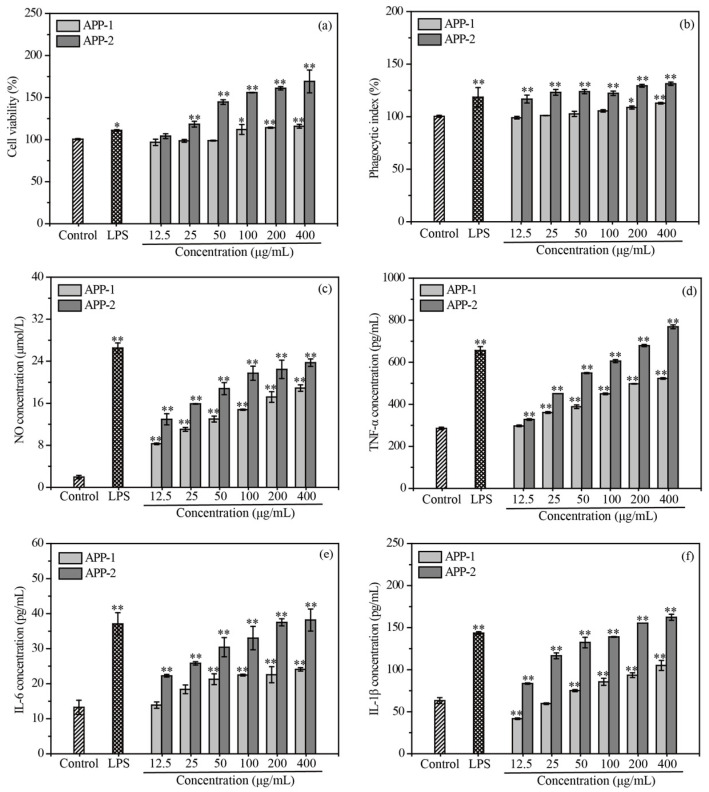
Effects of APP-1 and APP-2 on the viability (**a**), phagocytosis activity (**b**), secretions of NO (**c**), TNF-α (**d**), IL-6 (**e**) and IL-1β (**f**) of RAW 264.7 cells. * *p* < 0.05 vs. cell blank control, ** *p* < 0.01 vs. cell blank control.

The macrophages, as crucial mononuclear phagocytes, play a pivotal role in initiating and participating in the primary immune response, defending against pathogens and resisting cancer cell invasion [40]. The cytotoxicity of APP-1 and APP-2 on RAW 264.7 cells was assessed by performing a CCK-8 assay to determine cell viability. As shown in Figure 6a, compared to the control group, the cell viability of RAW 264.7 cells was significantly increased in a dose-dependent manner upon treatment with APP-2 (25–400 μg/mL). When APP-2 was at the lowest concentration of 12.5 μg/mL, the cell viability was 104.14%, and the cell viability could be up to 169.25% at 400 μg/mL APP-2 treatment, much exceeding the 1 μg/mL lipopolysaccharide (LPS) positive group. When RAW 264.7 cells were administrated APP-1, cell viability had no significant change within the concentration range of 12.5–50 μg/mL, but it gradually increased with an increase in the concentration range of 100–400 μg/mL. Therefore, the six gradient concentrations of APP-1 and APP-2 (12.5, 25, 50, 100, 200 and 400 μg/mL) were chosen in the follow-up experiments.

#### 2.6.2. Effects of APP-1 and APP-2 on the Phagocytosis Ability of RAW 264.7 Cells

Compared with non-activated macrophages, the phagocytosis of activated macrophages takes a crucial role in innate and adaptive immune responses, and it is also a significant indicator of immune evaluation [41]. A neutral red uptake experiment was conducted to assess the phagocytic effects of APP-1 and APP-2 on RAW 264.7 cells. The phagocytic rates of RAW 264.7 cells treated with different concentrations of APP-2 were significantly higher than those of the blank control group (*p* < 0.01), while a significant difference between APP-1 and the blank control group was observed only at concentrations of 200 and 400 μg/mL (Figure 6b). At the concentration of 400 μg/mL, both APP-1 and APP-2 obtained the maximum phagocytic rate of 112.80% and 131.30%, respectively. Furthermore, the phagocytosis index of APP-2 at the lowest concentration of 12.5 μg/mL was close to that of the LPS group. The results indicated that APP-1 and APP-2 could activate RAW 264.7 cells and enhance their phagocytic activity to different extents, and APP-2 especially was always superior to other groups within the concentration range of 12.5–400 μg/mL.

#### 2.6.3. Effects of APP-1 and APP-2 on the Secretions of NO and Cytokines

Activated macrophages are capable of generating a good deal of secondary compounds, such as NO, TNF-α, IL-6 and IL-1β, which play vital roles in activating various immune responses [42]. NO, a highly active intracellular messenger molecule, not only takes part in plentiful physiological and pathological activities but also mediates the destruction of pathogen and tumor cells by macrophages. Therefore, NO can be recognized as a quantitative indicator to judge the degree of macrophage activation [43]. As depicted in Figure 6c, compared with the control group, APP-1 and APP-2 significantly promoted RAW 264.7 cells to release NO (*p* < 0.01) in a dose-dependent manner at concentrations of 12.5–400 μg/mL. At a treatment concentration of 12.5 μg/mL, the NO production of APP-1 and APP-2 reached 8.29 and 12.96 μmol/L, respectively, which were 4.59 and 6.65 times higher than the control group. When the treatment concentration was 400 μg/mL, the NO release of the two eluted fractions reached the highest secretions of 18.89 and 23.73 μg/mL, respectively. It was worth noting that the order of NO concentration was always “APP-2 *>* APP-1” within the concentration range of 12.5–400 μg/mL. Abundant polysaccharides present in plants, algae or fungi could activate macrophages to produce NO. The NO generation of RAW 264.7 cells treated with 1000 μg/mL of the legume plant *Millettia Speciosa Champ* polysaccharide was only 10.90 μmol/L, which was lower than our research results [41]. In addition, the release of NO from RAW 264.7 cells was similar to LPS positive treatment.

Except for NO, activated macrophages also enhance the production of cytokines such as TNF-α, IL-6 and IL-1β, which can serve as important components of host defense and are closely involved in immune regulation, cell growth and the repair of injured tissue [44]. As shown in Figure 6d–f, the concentrations of TNF-α, IL-6 and IL-1β were gradually enhanced with the increase in sample concentration, which was in accordance with the findings of Li et al. [45]. Compared to the control group, lower concentrations of APP-1 did not significantly enhance the release of TNF-α, IL-6 and IL-1β by RAW 264.7 cells. However, APP-2 significantly increased the release of TNF-α, IL-6 and IL-1β across the entire experimental concentration range in 12.5–400 μg/mL. At the highest treatment concentration of 400 μg/mL, the release of TNF-α, IL-6 and IL-1β in APP-1 reached 523.12, 24.10 and 105.08 pg/mL, respectively, while the release of TNF-α, IL-6 and IL-1β in APP-2 reached 768.77, 38.18 and 162.36 pg/mL, respectively. Additionally, the secretion of TNF-α, IL-6 and IL-1β stimulated with APP-2 at the concentration of 200 μg/mL was even higher than the positive control LPS. The results showed that APP-2 had a greater stimulating effect on the secretions of the three cytokines than APP-1.

The immunomodulatory activity of polysaccharides was closely related to their composition and structure, including sulfate content, monosaccharide composition, Mw, connectivity patterns and sequences. It had been confirmed that polysaccharides with high levels of sulfate ions exhibited stronger immunomodulatory activity. As reported by Ferreira S. et al. [46], sulfated alpha-(1-4)-d-glucan exhibited strong immunostimulatory activity, while unsulfated alpha-(1-4)-d-glucan did not exert an immunostimulatory effect. High molecular weight polysaccharides had stronger immune regulatory activity, such as the purified component MSCP2 from *Millettia Speciosa* Champ polysaccharides, which could bind to more pattern recognition receptors (PPRs) on the surface of immune cells, promote inflammatory cytokines generation and enhance immune response attributed to its large Mw [41]. Likewise, the contents of glucose, arabinose, mannose and galactose were positively correlated with the generation of cytokines, which was due to PPRs preferring to combine with the aforementioned monosaccharides [47]. Given that APP-2 had strong antioxidant and immunoregulatory activity, it was considered for further structural characterization.

### 2.7. Characterization of APP-2

**Figure 7:**
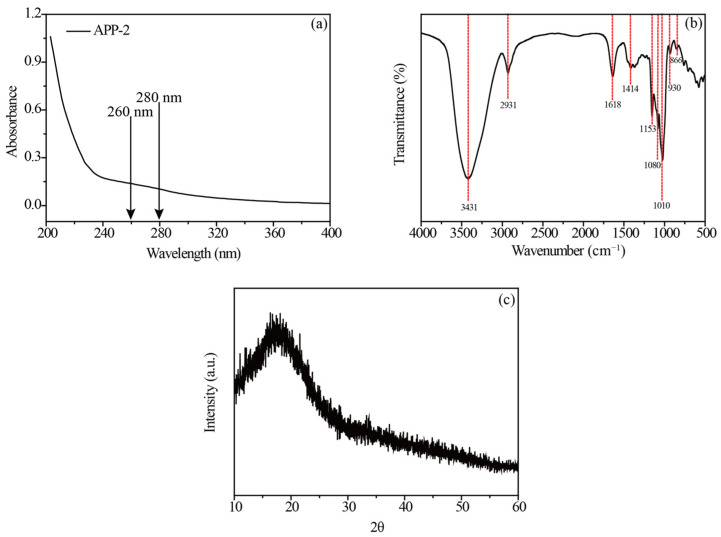
UV spectra (**a**), FTIR spectra (**b**) and XRD pattern (**c**) of APP-2.

The chemical composition of APP-2 is presented in Table 3, in which the contents of the total sugar and protein of APP-2 were 90.28% and 1.21%, respectively. Moreover, no obvious peaks were observed at 260 and 280 nm (Figure 7a), manifesting that nucleic acids and proteins did not exist in APP-2 and proving its high purity. Meanwhile, a sulfate content of 10.07%, a uronic acid content of 4.42% and a total phenolic content of 4.16% were attributed to APP-2, respectively. These results showed that APP-2 was acidic polysaccharide.

The HPSEC-MALLS-RI chromatogram (Figure S1a) of APP-2 displayed a single symmetrical and sharp peak, confirming the homogeneity of the sample. The Mw of APP-2 was 72.48 kDa, which differed from the polysaccharides isolated from *Spirulina platensis* extracted by Wu et al. [14]. It is worth mentioning that Mw differences resulted from the resources of the samples and test conditions [6]. In addition, the biological activities of different Mw components isolated from the same crude polysaccharides might have big differences, suggesting Mw directly influenced the functions of biological macromolecules, which was in line with previous literature about three polysaccharides fractions from *Rhodosorus* sp. SCSIO-45730 [48].

The monosaccharide compositions of APP-2 are shown in Table 3; it can be seen that APP-2 was a heteropolysaccharide consisting of rhamnose, glucose, mannose and glucuronic acid, in which the retention times of these four monosaccharides were 7.89, 11.96, 14.43 and 34.98 min (Figure S1b,c) and the mass percentages were 1.00:24.21:7.63:1.53 by comparing the spectra of the standard samples. Evidence suggested that the monosaccharide composition of *Spirulina* polysaccharides consisted of rhamnose, xylose, glucose, galactose, mannose and fucose in varying molar ratios, which might be due to the differences in source materials, processing methods, preparation procedures, etc. [6].

The FT-IR spectrum is widely employed to identify the functional groups of a sample. As observed in Figure 7b, the absorptions at 3431 and 2931 cm^−1^ were assigned to the stretching vibration of O-H and C-H, respectively, indicative of carbohydrate characteristic peaks [49]. In addition, there were absorption peaks at 1618 and 1414 cm^−1^ attributed to the asymmetric and symmetric stretching of –COO, which indicated the existence of uronic acids, in line with the monosaccharide composition analysis [50]. The signals at 1153 cm^−1^ indicated the presence of O=S=O [51]. Furthermore, a distinct absorption peak appeared at 1010 cm^−1^, characterizing the existence of the pyranose ring. Two small peaks at 930 and 866 cm^−1^ corresponded to beta and alpha configuration in APP-2 [2].

XRD has been commonly employed to investigate the crystalline properties of biological macromolecules. In most instances, broad diffraction peaks were typical of amorphous materials, while sharp peaks were characteristic of crystalline components [52]. Figure 7c showed that there was a broad and weak diffraction peak approximately at the 2θ of 18^◦^, manifesting that APP-2 had low overall crystallinity and mainly existed in an amorphous form, which corresponded to the study of Mo et al. [53].

**Figure 8:**
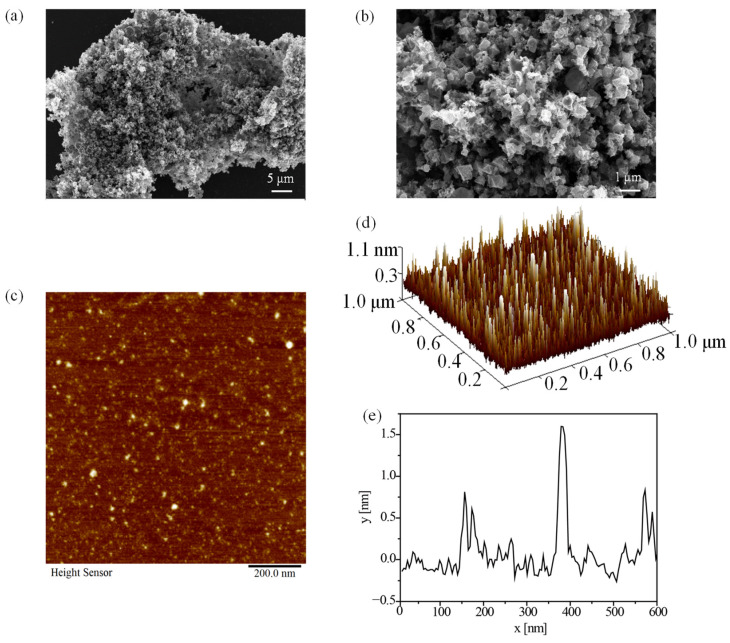
SEM images of APP-2 at 2000 (**a**) and 10,000 (**b**) magnifications. AFM images of APP-2 (**c**–**e**).

SEM is recognized as a strong instrument to explore the surface topographies of polysaccharides. The morphology structure of APP-2 was observed under magnifications of 2000× and 10,000×, shown in Figure 8a,b. APP-2 exhibited a cauliflower-like structure that consisted of granular and lamellar clusters irregularly folded, similar to the polysaccharides extracted from *Kangxian* flowers [2]. Moreover, the surface of APP-2 was smooth, which might result from the strong interactions between polysaccharide molecules [1].

AFM, as a high-resolution instrument, is usually adopted to illustrate the advanced structure of polysaccharide samples, such as nanostructure morphology, the length and diameter of the chain and the conformation of the chain [54]. Notably, APP-2 revealed linear and irregular spherical particle structures (Figure 8c,d), confirming the aggregation of polysaccharide molecules, which conformed to the structure of quinoa seeds polysaccharides and a purified polysaccharides component from *Rhodosorus* sp. SCSIO-45730 polysaccharides [48,55]. In addition, the roughness value of APP-2 was 0.14 nm, further verifying the observation in the SEM image. The height of APP-2 was 1.80 nm (Figure 8e). It is reported that the single-chain height of polysaccharides was approximately 0.1 to 1 nm, suggesting that the polysaccharide chains were branched, interweaved and tangled with each other. The phenomenon resulted from the presence of a mass of hydrogen bonds between sugar chains and van der Waals forces in polysaccharide molecules [56].

## 3. Materials and Methods

### 3.1. Materials and Reagents

Arthrospira platensis powder was provided by the Guangdong Province Economic Microalgae Germplasm Resource Bank (Guangzhou, China). The RAW 264.7 cell was obtained from Wuhan Pricella Biotechnology Co., Ltd. (Wuhan, China). DEAE-52 cellulose, Sephadex G-100, DPPH, ABTS, CCK-8 and LPS were purchased from Sigma Chemical Co. (St. Louis, MO, USA). NO quantitation kits were purchased from Nanjing Jiancheng Biological Engineering Institute (Nanjing, China). Beyotime Institute of Biotechnology Co., Ltd. (Shanghai, China) provided TNF-α, IL-6 and IL-1β ELISA kits. Thirteen monosaccharide standards, including fucose, rhamnose, arabinose, galactose, glucose, xylose, mannose, fructose, ribose, galacturonic acid, guluronic acid, glucuronic acid and mannuronic acid were purchased from Sigma-Aldrich (St. Louis, MO, USA). All reagents used in the study were of analytical grade.

### 3.2. UAE of APP

Firstly, the material was crushed and sieved by a 60-mesh sieve. Secondly, 80% ethanol (raw material: 80% ethanol = 1:10 g/mL) was added to Arthrospira platensis powder to remove lipids and pigments. The mixtures were rotated continuously at room temperature, centrifuged at 6876× *g* for 15 min and decolorization was repeated three times. Then, the algal sludge was lyophilized, ground into powder and stored for subsequent utilization. APP was extracted by the modified UAE method reported by Zhang et al. [19]. In brief, 150 mL of deionized distilled water was added to 5 g of algal tissue and the resultant solution was kept at room temperature for 2 h before being extracted by an ultrasonic bath (KQ-400KDE, Kunshan Ultrasonic Instrument Co., Ltd., Kunshan, China) at 60 °C. The ultrasonic power was 150 W for 30 min. Many factors can affect the effectiveness of extraction while using UAE, such as the ultrasonic source, frequency and intensity, extraction time and temperature, the choice of solvent and the solid–liquid ratio [57]. A single-factor experiment was conducted for four UAE parameters, including the liquid–solid ratio (10–50 mL/g), extraction temperature (50–90 °C), ultrasonic power (60–180 W) and extraction time (20–60 min), based on common sense after consulting literature that adopted ultrasound to extract polysaccharides from Imperata cylindrica [58], maidenhairtree [59], prickly pear peels [60], Sargassum angustifolium [61] and Porphyra yezoensis [62], in which the APP yield was used as the evaluation index. Each test was conducted in triplicates. The crude extract was centrifuged at 10,744× *g* for 15 min and the supernatant was concentrated by vacuum rotary evaporation at 50 °C, as well as with the use of 3-fold alcohol sedimentation, Savage method (chloroform/butanol, 5/1, *v*/*v*) deproteinization, dialysis (Mw 500 Da cutoff) and freeze-drying to yield the crude APP. The extraction yield (Y) of crude APP was calculated using Equation (2):

Y(%,*w*/*w*) = (weight of APP/weight of *Arthrospira platensis*) × 100% (2)

### 3.3. Response Surface Optimization

Based on the results of the single-factor experiment (Figure 1), the BBD employed in RSM involved four variables: a liquid–solid ratio (A) of 10, 20 and 30 mL/g, extraction temperature (B) of 70, 80 and 90 °C, ultrasonic power (C) of 60, 90 and 120 W and extraction time (D) of 20, 30 and 40 min. Each variable had three different levels (−1, 0 and 1) and APP yield (Y) served as the response. The Design Expert Software (8.0.6) was adopted to devise and analyze regression fitting on four factors to determine the optimization of extraction parameters. Moreover, the accuracy of the statistical experimental strategy was validated by conducting three additional confirmatory extraction experiments under optimal conditions [33].

### 3.4. Separation and Purification of APP

For further purification, freeze-dried crude polysaccharides were re-dissolved in distilled water to a concentration of 20 mg/mL and then loaded onto a pre-equilibrated Cellulose DEAE-52 column (3.5 cm × 40 cm) (Sigma Chemical Co., St. Louis, MO, USA). The DEAE-52 cellulose column chromatography facilitated the efficient isolation and purification of polysaccharides due to its exceptional ion-exchange properties, particularly the high adsorption capacity for ionic species. The column was eluted with distilled water and a series of NaCl solutions (0.3, 0.5, 0.8 and 1.2 mol/L, respectively) at a flow rate of 2 mL/min. Each eluted fraction (10 mL/tube) was individually collected and subjected to detection using the phenol–sulfuric acid method [63]. Subsequently, eluates from the same predominantly major peak were combined, concentrated, dialyzed and lyophilized to yield two major elution fractions named APP-1 and APP-2, respectively. On this basis, a 500 mg sample was re-dissolved in 10 mL of distilled water, followed by centrifugation at 10,744× *g* for 15 min. The resulting solution was filtered through a 0.45 um membrane, and then the dissolved sample was loaded onto the Sephadex G-100 column (Sigma Chemical Co., St. Louis, MO, USA) with distilled water as eluent (at a flow rate of 0.3 mL/min) [64]. Sephadex G-100 gel column chromatography is an effective method based on the interaction of molecular sieves that can be used for the separation of polysaccharides. Finally, the eluent with a good single peak was collected, pooled, concentrated, dialyzed and freeze-dried to obtain the final product for the following experiments.

### 3.5. Characterization of APP-2

#### 3.5.1. Chemical Composition Analysis

The total carbohydrate content of APP-2 was analyzed by the phenol–sulfuric acid method with glucose as a standard [63]. The protein content was determined using the Lowry method with bovine serum albumin (BSA) as the standard [8]. A BaCl_2_-gelatin turbidity assay was employed to measure the sulfate content of APP-2 [33]. The uronic acid content of APP-2 was analyzed by the meta-hydroxydiphenyl assay with GalA as a standard at 520 nm [65]. The total phenolic content (TPC) of APP-2 was determined using the Folin–Ciocalteu method, based on the calibration curve established for gallic acid [66].

#### 3.5.2. Distribution of Average Molecular Weight

The homogeneity and average molecular weight of APP-2 were measured by HPSEC instrument equipped with a multi-angle laser light scattering photometer (MALLS) and a refractive index detector (RID) (Optilab T-rEX, Wyatt Technology Co., Santa Barbara, CA, USA). The detailed procedure is described in Supplementary Methods S1.

#### 3.5.3. Monosaccharide Composition Analysis

The monosaccharide composition of APP-2 was measured by HPAEC (Thermo Fisher Scientific, Waltham, MA, USA) with 13 monosaccharide standards. A detailed process is provided in Supplementary Methods S2.

#### 3.5.4. Spectroscopic Methods and FT-IR Assay

APP-2 was dissolved in distilled water (1 mg/mL) and its absorbance was recorded using a TU-1810 spectrophotometer (Beijing Persee General Instrument Co., Ltd., Beijing, China) in the spectral scanning range of 200−400 nm^−1^ to identify the presence of nucleic acids and proteins in the sample [67]. FT-IR (IR Affinity-1, Shimadzu, Japan) was employed to determine the organic functional groups of APP-2 at a wavenumber of 400 cm^−1^–4000 cm^−1^. The scan number was 32.

#### 3.5.5. XRD

XRD analysis was conducted at room temperature using Cu Kα radiation on a MXP18 HF diffractometer (MAC Science Co., Yokohama, Japan) and the patterns were collected between 10 and 90° at a scanning rate of 10.0°/min.

#### 3.5.6. SEM

SEM (Sigma 300/VP, Zeiss, Jena, Germany) was adopted to observe the surface characterization and microstructure evaluation of APP-2 at a voltage of 3.0 kV. The freeze-dried APP-2 sample was securely affixed to a sample holder using double-sided carbon tape, meticulously coated with a thin layer of gold, and subsequently subjected to analysis within a high vacuum chamber.

#### 3.5.7. AFM

The ultrastructural features of APP-2 were visualized by a scanning probe microscope (Bruker, Karlsruhe, Germany). The sample was dissolved in distilled water at a concentration of 1 μg/mL, and subjected to ultrasonication for 30 min, followed by the dispersion of 2–3 µL of APP-2 solution onto cleaved micas. Subsequently, an analysis was performed after drying at 120 °C for 0.5 min [68]. The height of APP-2 was determined by the Nanoscope software (1.7).

### 3.6. Antioxidant Activity Assessment

The DPPH, ABTS, hydroxyl free radicals scavenging activity and ferrous irons chelating ability of APP-1 and APP-2 were modified as previously reported [2,69]. Supplementary Methods S3 provides the detailed procedures.

### 3.7. Immunomodulatory Activity Assay

#### 3.7.1. RAW 264.7 Cultivation

RAW 264.7 cells were cultured in Dulbecco’s modified Eagle medium (DMEM) containing 10% fetal bovine serum and 1% penicillin–streptomycin under a 5.0% CO_2_ incubator at 37 °C. Upon reaching a convergence level of 80–90%, the cells were trypsinized, centrifuged at 107× *g* for 4 min and resuspended in fresh DMEM medium for further tests.

#### 3.7.2. Evaluation of Cell Viability

Cell viability was measured by CCK-8 assay [70]. Briefly, RAW 264.7 cells were in the exponential/linear phase and seeded in 96-well plates at a density of 2 × 10^4^ cells per well at 37 °C for 24 h with 5% CO_2_. After that, 100 μL of APP-1 or APP-2 solution prepared in DMEM was added to reach the final concentrations (12.5, 25, 50, 100, 200 and 400 μg/mL) and the cells were cultured in the incubator for another 24 h. Cells only treated with 1.0 μg/mL LPS were set as the positive group and the untreated macrophages in complete medium were set as another control group. Then, a volume of 10 μL CCK-8 was added to each well and incubated for 1–4 h. The absorbance was recorded at 450 nm using a Multiskan Sky microplate reader (Thermo Fisher Scientific, Waltham, MA, USA). The computation formula of cell viability was as follows:

Cell viability (%) = (A_sample_/A_control_) × 100 (3)

where A_sample_ and A_control_ represented the values of the experimental group and the control group, respectively.

#### 3.7.3. Analysis of Cell Phagocytosis

The effects of APP-1 and APP-2 on the phagocytic activity of RAW 264.7 cells were determined by the neutral red staining approach. After treating RAW 264.7 cells with samples or LPS for 24 h, the supernatants were discarded and each well was supplemented with 100 μL of a 0.1% neutral red solution, followed by a 30 min incubation period. Cells were washed three times with PBS to move free neutral red and treated with 100 μL of cell lysate buffer (acetic acid: ethanol acid = 1:1, *v*/*v*) for the whole night at room temperature. The absorbance was determined at 540 nm.

#### 3.7.4. Determination of NO and Cytokines (TNF-α, IL-6 and IL-1β)

RAW 264.7 cells were treated as described for the cell viability assay and the supernatants of different treatment groups were collected. The quantitative analysis of NO, TNF-α, IL-6 and IL-1β was conducted in accordance with the instructions provided by the ELISA kits.

### 3.8. Statistical Analysis

All experiments were repeated a minimum of three times and the results were expressed as mean and standard deviations. Statistical analysis was conducted using Design Expert Software (8.0.6) and IBM SPSS Statistics 25 (SPSS Inc., Chicago, IL, USA) software. *p <* 0.05 was regarded as statistically significant. Origin Software Version 2010 (Origin Lab Corp., Northampton, MA, USA) was adopted to draw all graphs.

## 4. Conclusions

In this research, the UAE conditions of APP were optimized by RSM to improve its extraction rate. The optimized UAE conditions were as follows: a liquid–solid ratio of 30.00 mL/g; extraction temperature of 81 °C; ultrasonic power of 92 W and extraction time of 30 min, under which the maximum extraction yield of APP reached 14.78%. Afterward, two polysaccharides (APP-1 and APP-2) were isolated and purified from the crude polysaccharides extracted from *Arthrospira platensis*. APP-1 and APP-2 all possessed potential antioxidative and immunoregulatory properties. However, among the two polysaccharides samples, APP-2 displayed more significant scavenging activities against DPPH (IC_50_ = 0.27 mg/mL), ABTS (IC_50_ = 0.97 mg/mL) and hydroxyl radicals (IC_50_ = 1.47 mg/mL), as well as ferrous chelating ability (IC_50_ = 1.23 mg/mL), than APP-1. On the other hand, APP-2 showed more pronounced immune activity, by amplifying phagocytosis and promoting the secretions of NO and all kinds of cytokines like TNF-α, IL-6 and IL-1β, than APP-1. Meanwhile, the chemical composition and basic characterization of APP-2 were identified by HPSEC, HPAEC, UV-Vis, FT-IR, XRD, SEM and AFM. The results indicated that the Mw of APP-2 was 72.48 kDa, which contained rhamnose, glucose, mannose and glucuronic acid at a ratio of 1.00:24.21:7.63:1.53, respectively. Notably, APP-2 was found to present an amorphous cauliflower-like structure consisting of granular and lamellar clusters with irregular folding. It could be supposed that the strong antioxidant and immunomodulatory activities of APP-2 were closely linked to its low Mw, uronic acid content, sulfate content and monosaccharide composition, linkage patterns and sequences. Therefore, ultrasonic treatment effectively obtained polysaccharides from *Arthrospira platensis* with great potential in functional food and pharmaceutical products. However, further research, including in vivo activity experiments, is required to develop economical and environmentally friendly extraction and purification techniques for APP, reveal a more detailed structure–function relationship, and reveal the pharmacokinetic characteristics of APP in order to strengthen the development of functional food and pharmaceutical products containing APP.

## Figures and Tables

**Table 1:** Box–Behnken design matrix with coded variables and experimental and predicted values of response.

Run	Liquid–Solid Ratio (mL/g)	Extraction Temperature (°C)	Ultrasonic Power (W)	Extraction Time (min)	Polysaccharide Yield (%)	Predicted Value (%)
1	0 (20)	0 (80)	0 (90)	0 (30)	14.3 ± 0.11	14.1
2	−1 (10)	1 (90)	0 (90)	0 (30)	11.7 ± 0.24	12.1
3	0 (20)	1 (90)	0 (90)	−1 (20)	9.0 ± 0.12	9.3
4	0 (20)	−1 (70)	−1 (60)	0 (30)	6.8 ± 0.04	7.3
5	−1 (10)	0 (80)	0 (90)	−1 (20)	12.8 ± 0.02	13.3
6	1 (30)	0 (80)	1 (120)	0 (30)	12.0 ± 0.11	12.4
7	0 (20)	−1 (70)	0 (90)	1 (40)	7.4 ± 0.25	7.4
8	1 (30)	0 (80)	−1 (60)	0 (30)	11.3 ± 0.22	11.9
9	0 (20)	−1 (70)	1 (120)	0 (30)	7.0 ± 0.20	7.9
10	−1 (10)	0 (80)	1 (120)	0 (30)	13.1 ± 0.03	12.7
11	0 (20)	1 (90)	−1 (60)	0 (30)	8.8 ± 0.25	8.2
12	1 (30)	1 (90)	0 (90)	0 (30)	12.7 ± 0.03	12.1
13	0 (20)	0 (80)	0 (90)	0 (30)	14.1 ± 0.06	14.1
14	−1 (10)	−1 (70)	0 (90)	0 (30)	10.4 ± 0.13	10.4
15	1 (30)	0 (80)	0 (90)	1 (40)	13.3 ± 0.16	13.1
16	0 (20)	0 (80)	1 (120)	1 (40)	11.8 ± 0.13	11.6
17	1 (30)	0 (80)	0 (90)	−1 (20)	12.7 ± 0.20	13.4
18	0 (20)	0 (80)	0 (90)	0 (30)	13.9 ± 0.03	14.1
19	0 (20)	0 (80)	1 (120)	−1 (20)	10.0 ± 0.12	9.4
20	0 (20)	1 (90)	0 (90)	1 (40)	10.2 ± 0.02	10.9
21	0 (20)	0 (80)	−1 (60)	−1 (20)	11.3 ± 0.21	11.0
22	0 (20)	1 (90)	1 (120)	0 (30)	10.0 ± 0.12	9.8
23	−1 (10)	0 (80)	0 (90)	1 (40)	13.0 ± 0.04	12.6
24	−1 (10)	0 (80)	−1 (60)	0 (30)	11.1 ± 0.19	10.9
25	0 (20)	0 (80)	−1 (60)	1 (40)	7.7 ± 0.02	7.8
26	0 (20)	−1 (70)	0 (90)	−1 (20)	10.5 ± 0.19	10.0
27	1 (30)	−1 (70)	0 (90)	0 (30)	12.0 ± 0.03	11.1

**Table 2:** Analysis of variance for regression model of polysaccharide extraction yield from *Arthrospira platensis*.

Source	Coefficient	Sum of Squares	df	Mean Square	F-Value	*p*-Value
Model	14.10	118.50	14	8.46	17.24	<0.0001
A	0.16	0.30	1	0.30	0.61	0.4490
B	0.69	5.74	1	5.74	11.69	0.0051
C	0.57	3.97	1	3.97	8.08	0.0148
D	−0.24	0.70	1	0.70	1.43	0.2553
AB	−0.15	0.090	1	0.090	0.18	0.6762
AC	−0.33	0.42	1	0.42	0.86	0.3719
AD	0.10	0.040	1	0.040	0.081	0.7802
BC	0.25	0.25	1	0.25	0.51	0.4892
BD	1.07	4.62	1	4.62	9.41	0.0098
CD	1.35	7.29	1	7.29	14.84	0.0023
A^2^	0.52	1.18	1	1.45	2.95	0.1118
B^2^	−3.18	55.61	1	53.90	109.76	<0.0001
C^2^	−2.63	38.28	1	36.87	75.07	<0.0001
D^2^	−1.53	13.30	1	12.47	25.39	0.0003
Residual		5.89	12	0.49		
Lack of Fit		5.81	10	0.48	14.53	0.0661
Pure Error		0.080	2	0.040		
Cor Total		124.40	26			
C.V.% Adeq precision	6.33 13.066					
R^2^ Adj- R^2^	0.9526 0.9002					

**Table 3:** Chemical composition of APP-2.

Index	Values
Total sugar (%)	90.28 ± 0.32
Protein (%)	1.21 ± 0.03
Sulfate (%)	10.07 ± 0.14
Uronic acid (%)	4.42 ± 0.67
Total phenolic (%)	4.16 ± 0.03
Mw (kDa)	72.48
Monosaccharide composition (%)	
Rhamnose	1.00
Glucose	24.21
Mannose	7.63
Glucuronic Acid	1.53

## Data Availability

The original contributions presented in the study are included in the article and Supplementary Materials; further inquiries can be directed to the corresponding author.

## Supplementary Materials

The following supporting information can be downloaded at: https://www.mdpi.com/article/10.3390/molecules29194645/s1, Figure S1: HPSEC-MALLS-RI profiles of APP-2 (a), ion chromatography profiles of mixed standard monosaccharides (b) and monosaccharide compositions of APP-2 (c). The peaks in chromatography profile (b) from left to right are as follows: (1) fucose; (2) rhamnose; (3) arabinose; (4) galactose; (5) glucose; (6) xylose; (7) mannose; (8) fructose; (9) ribose; (10) galacturonic acid; (11) guluronic acid; (12) glucuronic acid and (13) mannuronic acid.; Table S1: IC_50_ of APP-1 and APP-2 in antioxidant activity assays. Supplementary Methods S1: Homogeneity and molecular weight distribution analysis; Methods S2: Monosaccharide composition analysis; Methods S3: Antioxidant activity assessment. References [1,2,70,71,72,73] are cited in the Supplementary Materials.
