# Supplementary material for: Optimization of Ultrasonic-Assisted Extraction, Characterization and Antioxidant and Immunoregulatory Activities of Arthrospira platensis Polysaccharides
Source: Molecules. 2024 Sep 30;29(19):4645. doi: 10.3390/molecules29194645 (PMC11477882; doi:10.3390/molecules29194645)
Supplement: Supplementary file 1 [file molecules-29-04645-s001.zip › molecules-3197211-supplementary.pdf]

Optimization of Ultrasonic-Assisted Extraction, Characterization and Antioxidant and Immunoregulatory Activities of *Arthrospira platensis* Polysaccharides

Na Wang [1,2], Jingyi Qin [2], Zishuo Chen [3,4], Jiayi Wu [3,5] and Wenzhou Xiang [3,*]

1 Department of Cell Biology and Genetics, School of Basic Medical Sciences, Hengyang Medical School, University of South China, Hengyang 421001, China
2 Institute of Cytology and Genetics, School of Basic Medical Sciences, Hengyang Medical School, University of South China, Hengyang 421001, China
3 CAS Key Laboratory of Tropical Marine Bio-Resources and Ecology, Guangdong Key Laboratory of Marine Materia Medica, Institution of South China Sea Ecology and Environmental Engineering, RNAM Center for Marine Microbiology, South China Sea Institute of Oceanology, Chinese Academy of Sciences, Guangzhou 510301, China
4 University of Chinese Academy of Sciences, Beijing 100049, China
5 Greater Bay Area Institute of Precision Medicine (Guangzhou), Guangzhou 511466, China
* Correspondence: xwz@scsio.ac.cn; Tel.: +86-020-8902-3223

Supplementary methods

S1 Homogeneity and molecular weight distribution analysis

Molecular weight (Mw), as a fundamental property of polysaccharides, plays a crucial role in the structural and functional biological activities of polysaccharides. The homogeneity and Mw distribution of RSP were determined by High-performance size-exclusion chromatography-multi angle laser light scattering-refractive index detector (HPSEC-MALLS-RI). The basic light-scattering equation is as follows:

$$\frac{Kc}{R_\theta} = \frac{1}{Mw}\left[1 + \frac{16\Pi^2 n^2}{3\lambda_0^2}<s^2>_z \sin^2\left(\frac{\theta}{2}\right)\right] + 2A_2 c + \ldots \quad (S1)$$

$$K = \frac{4\Pi^2 n^2}{N_A \lambda_0^4}\left(\frac{dn}{dc}\right)^2 \quad (S2)$$

where n is the refractive index of the solvent; K is the optical constant; λ_0 is the wavelength of the laser in a vacuum; N_A is the Avogadro's number; R_θ is the Rayleigh ratio; A_2 is the second viral coefficient; c is the mass concentration; dn/dc is the refractive index increment. A differential refractive index detector (Optilab T-rEX, Wyatt Technology Co., Santa Barbara, CA, USA) was simultaneously connected to give the concentration of fractions and the dn/dc value. Data were acquired and processed using ASTRA6.1 (Wyatt Technology).

S2 Monosaccharide composition analysis

13 monosaccharides including fucose, rhamnose, arabinose, galactose, glucose, xylose, mannose, fructose, ribose, galacturonic acid, glucuronic acid, mannuronic acid and guluronic acid were chosen as the external standards. Approximately 5 mg of sample was hydrolyzed with trifluoroacetic acid (2 M) at 60 °C for 30min in a sealed tube, followed by nitrogen blow to dry. Add methanol to wash, then blow-dry, repeat

methanol wash 2-3 times. The residue was redissolved in deionized water and filtered through 0.22 μm microporous filtering film for testing.

S3 Antioxidant activity assessment

DPPH radical scavenging activity

The DPPH scavenging assay is a vital instrument to test the antioxidant activity of bioactive compounds. A fresh DPPH methanolic solution (0.1 mM, dissolved in 95% ethanol) was prepared. Then, 100 μL of DPPH methanolic solution was added to the 100 μL polysaccharides fractions (APP-1 and APP-2) at different concentrations (0.125, 0.25, 0.5, 1.0 and 2.0 mg/mL) and the mixtures were vortexed for 1 min and stored at 25°C in the dark for 30 min. The absorbance was measured at 517 nm and ascorbic acid (Vc) was used as reference standard [1]. The ability to scavenge the DPPH radical was calculated as follows:

$$\text{DPPH scavenging rate (\%)} = [1-(A_{sample}-A_{sample\ blank})/A_{control}] \times 100 \quad \text{(S3)}$$

Where DPPH solution plus APP sample was used as A_{sample}, APP sample without DPPH solution was used as $A_{sample\ blank}$, and DPPH solution without APP sample was used as $A_{control}$.

ABTS radical scavenging activity

ABTS radical scavenging has also been commonly used to assess the antioxidant effect of compounds. ABTS radical cation solution was prepared by a 12-16 h reaction of ABTS (7.4 mM) with potassium persulfate (2.6 mM) in a volume ratio of 1:1 at room temperature in the dark. The prepared ABTS solution was diluted 20.3 times with H_2O to an absorbance of 0.70 ± 0.02 at 734 nm. Then, 0.2 mL samples in

the concentration range of 0.125-2.0 mg/mL were mixed with 0.8 mL diluted ABTS. Shaking the mixture for 10 s, letting it stand for 6 min and finally pipetting 200 μL to record the absorbance at 734 nm [2]. Vc was set as a positive control. The activity to scavenge ABTS radical was calculated with the formula below:

ABTS scavenging rate (%) = $[1-(A_{sample}-A_{sample\ blank})/A_{control}] \times 100$ (S4)

Where A_{sample} contained ABTS solution and APP solution, $A_{sample\ blank}$ contained APP solution and H_2O, and $A_{control}$ contained ABTS solution and H_2O.

Hydroxyl radical scavenging activity

Hydroxyl radicals are by-products of biological, photochemical and immune actions, which can pass through the cell membrane at specific locations and react with biological macromolecules, leading to human aging and cell damage [3]. Thus, the removal of hydroxyl radicals is highly significant. The hydroxyl radical scavenging capacity of APP was analyzed by the method of Chen et al. [4] with some modifications. Briefly, 1.0 mL of 9.0 mM $FeSO_4$ and 1.0 mL of 9.0 mM ethanol salicylate were added to 1.0 mL APP of different concentrations (0.125, 0.25, 0.5, 1.0 and 2.0 mg/mL) in 5 mL test tubes, then in which 1.0 mL of 9.0 mM H_2O_2 was added. The mixtures were reacted at 37 °C for 30 min and then the absorbance was measured at 510 nm. Vc served as the positive control. The ability to scavenge the hydroxyl radical was calculated using the following equation:

Hydroxyl radical scavenging rate (%) = $[1-(A_{sample}-A_{sample\ blank})/A_{control}] \times 100$ (S5)

Here, A_{sample} and $A_{control}$ were the absorbance of the tested samples and the control group (prepared with distilled water), respectively. $A_{sample\ blank}$ was the absorbance of

the reagent blank (water in place of H_2O_2).

Ferrous ion chelating activity

Ferrous ions can produce reactive oxygen species through Fenton free radical reaction, accelerating lipid oxidation rates and causing oxidative damage to cells [5]. Hence, the ferrous ion-chelating assay has been generally used to assess the antioxidant activity of various polysaccharides. 0.5 mL of the polysaccharides solution at different concentrations (0.125, 0.25, 0.5, 1, 2 mg/mL) was mixed with 0.5 mL of $FeCl_2$ solution (2 mM) and ferrozine solution (5 mM). The mixtures were vortexed for 1 min and incubated for 10 min at 25 ◦C before being measured at 562 nm [6]. EDTA-2Na was used as standard. The chelating ability of ferrous ions was calculated by the following formula:

Ferrous ion chelating ability (%) = $[1-(A_{sample}-A_{sample\ blank})/A_{control}] \times 100$ (S6)

Where A_{sample} is the absorbance of the reaction mixture, $A_{sample\ blank}$ is the reagent without APP sample, and $A_{control}$ is the absorbance of APP sample.

Supplementary figures and tables

Figure S1

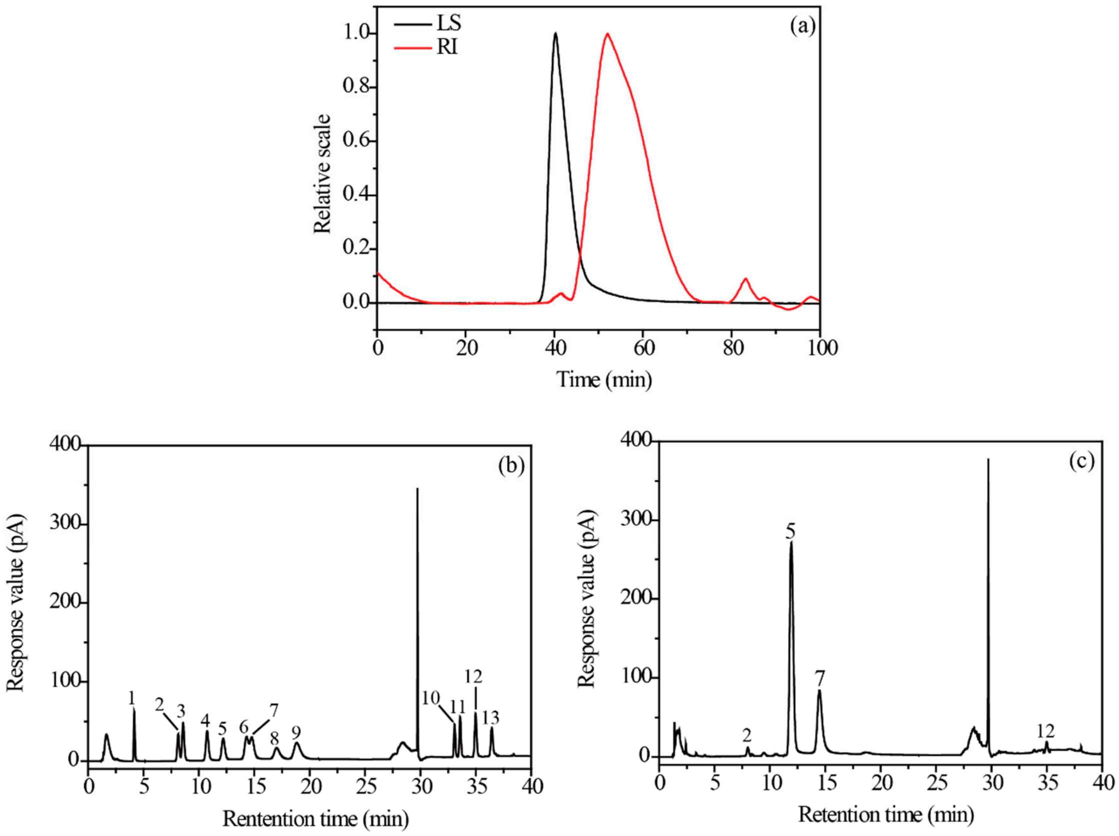

Figure S1 HPSEC-MALLS-RI profiles of APP-2 (a), Ion chromatography profiles of mixed standard monosaccharides (b), and monosaccharide compositions of APP-2 (c). The peaks in chromatography profile (b) from left to right order are as follows: (1) Fucose; (2) Rhamnose; (3) Arabinose; (4) Galactose; (5) Glucose; (6) Xylose; (7) Mannose; (8) Fructose; (9) Ribose; (10) Galacturonic Acid; (11) Guluronic Acid; (12) Glucuronic Acid; (13) Mannuronic Acid.

Table S1

Table S1. IC_{50} of APP-1 and APP-2 in antioxidant activity assays

IC_{50} (mg /mL)	Sample			
	APP-1	APP-2	Ascorbic acid	EDTA-2Na
DPPH	6.03	0.27	6.60E-5	-
ABTS	8.85	0.97	0.06	-
·OH	26.05	1.47	0.14	-
Ferrous chelation	3.75	1.23	-	0.07

References

1. Zhang, Y.; Liu, Y.-H.; Cai, Y.-Y.; Tian, Y.-P.; Xu, L.-F.; Zhang, A.-B.; Zhang, C.; Zhang, S.-S., Ultrasonic-assisted extraction brings high-yield polysaccharides from *Kangxian* flowers with cosmetic potential. *Ultrason. Sonochem.* 2023, 100, 106626. https://doi.org/10.1016/j.ultsonch.2023.106626.
2. Peng, Y.; Zhu, X.-C.; Yang, G.-Y.; Zhang, J.-J.; Wang, R.; Shen, Y.-B.; Li, H.-M.; Gatasheh, M.-K.; Abbasi, A.-M.; Yang, X.-Q., Ultrasonic extraction of *Moringa oleifera* seeds polysaccharides: optimization, purification, and anti-inflammatory activities. *Int. J. Biol. Macromol.* 2024, 258, 128833. https://doi.org/10.1016/j.ijbiomac.2023.128833.
3. Song, H.; He, M.; Gu, C.; Wei, D.; Liang, Y.; Yan, J.; Wang, C. Extraction optimization, purification, antioxidant activity, and preliminary structural characterization of crude polysaccharide from an arctic *Chlorella* sp. *Polymers* 2018, 10, 292. https://doi.org/10.3390/polym10030292.
4. Chen, Y.-X.; Liu, X.-Y.; Xiao, Z.; Huang, Y.-F.; Liu, B., Antioxidant activities of polysaccharides obtained from *Chlorella pyrenoidosa via* different ethanol concentrations. *Int. J. Biol. Macromol.* 2016, 91, 505-509. https://doi.org/10.1016/j.ijbiomac.2016.05.086.
5. Wang, S.; Li, G.; Zhang, X.; Wang, Y.; Qiang, Y.; Wang, B.; Zou, J.; Niu, J.; Wang, Z. Structural characterization and antioxidant activity of *Polygonatum sibiricum* polysaccharides. *Carbohydr. Polym.* 2022, 291, 119524. https://doi.org/10.1016/j.carbpol.2022.119524.
6. Alencar, P-.O.-C.; Lima, G.-C.; Barro, F.-C.-N.; Costa, L.-E.-C.; Ribeiro, C.-V.-P.-E.; Sousa, W.-M.; Sombra, V.-G.; Abreu, C.-M.-W.-S.; Abreu, E.-S.; Pontes, E.-O.-B.; Oliveira, A.-C.; de Paula, R.-C.-M.; Freitas, A.-L.-P., A novel antioxidant sulfated polysaccharide from the algae *Gracilaria caudata*: in vitro and in vivo activities. *Food Hydrocoll.* 2019, 90, 28-34. https://doi.org/10.1016/j.foodhyd.2018.12.007.
